# Design and synthesis of bio-based UV curable PU acrylate resin from itaconic acid for coating applications

**DOI:** 10.1080/15685551.2016.1231045

**Published:** 2016-10-25

**Authors:** Deepak M. Patil, Ganesh A. Phalak, S. T. Mhaske

**Affiliations:** ^a^ Department of Polymer and Surface Engineering, Institute of Chemical Technology, Mumbai, India

**Keywords:** Sustainable resource, itaconic acid, bio-based, PU acrylate resin, UV curable, coating application

## Abstract

UV curable PUA resin was successfully synthesized from polyol based on sustainable resource originated from itaconic acid (IA), isophorone diisocyanate (IPDI) and 2-hydroxyethyl methacrylate (HEMA). A polyol was synthesized by condensation reaction of IA with 16-hexanediol in the presence of p-Toluenesulfonic acid (pTSA). The synthesized PUA resin was characterized for its structural elucidation by using Fourier Transform Infrared Spectrophotometer (FTIR), ^1^H and ^13^C NMR spectroscopy. The synthesized UV curable PUA resin was incorporated in varying concentrations in conventional PUA coating system. The effects of varying concentration of synthesized UV curable PUA resin on rheology, crystallinity, thermal and coating properties were evaluated. The rheological behavior of the resins were evaluated at variable stress and result showed decrease in viscosity of resin as concentration of synthesized UV curable PUA resin increases in conventional PUA resin. The cured coatings have been evaluated for glass transition temperature (*T*_g_) and thermal behavior by differential scanning calorimeter and thermogravimetric analysis respectively. The degree of crystallinity of the coatings was determined from X-ray diffraction patterns using the PFM program. It was found that increase in the mass proportion of IA based PUA in coatings, the coating becomes more rigid and crystalline. The synthesized UV curable PUA coatings showed interesting mechanical, chemical, solvent and thermal properties as compared to the conventional PUA. Further, cured coatings were also evaluated for gel content and water absorption.

## 1. Introduction

The coatings are widely used for various purposes such as decoration, protection, and some specific functions. The various types of polymeric resins such as epoxy, polyurethane, polyester and acrylate was used to formulate the coatings. To gain the application viscosity volatile organic chemicals (VOCs) are added in most coating formulations, which can be evaporated, and transport to atmosphere at ambient conditions. Such materials have been making many pollution risks such as: their ability to forming undesired photochemical ozone smog, and the potential to cause carcinogenic and mutagenic.[1,2] Environmental concerns and health related issue have stimulated researcher to develop environment friendly coatings with low or no VOC content.[3] To fulfill these requirements, researcher developed various type of coating such as high solid, water dispersible,[4] powder,[5] radiation (UV/EB) curing coatings.[6,7] Among them, UV-curing technology has been commonly used in various industrial sectors to achieve an ultrafast hardening of protective coatings, printing inks, adhesives, varnishes and composites. Compared with traditional thermal curing, UV-curing technology is becoming more and more acceptable for its various advantages, such as less organic solvent, shorter reaction time, lower energy consumption, and being environmental friendly and efficiency.[8,9] Nowadays, UV-curable resins, such as epoxy acrylate, polyurethane acrylate (PUA), and acrylic resin, are available in the market. Among the various UV-curable resins, PUA has attracted great interest in the coatings and adhesives due to its unique properties, such as outstanding adhesion, excellent flexibility and chemical resistance. PUA resins are usually prepared by the reaction of a polyol with a diisocyanates to yield an isocyanate terminated oligomer and subsequently isocyanate terminated groups utilized to react with hydroxyl functional acrylic monomers incorporated the unsaturation at the end of the polymer backbone.[10]

Further, Fossil reserves are expected to deplete by the year 2050 if their present consumption rate continues.[11] For this reason, there is an increasing interest in the utilization of bio-based feedstock in both academic and industry laboratories. The increasing worldwide interest for the use of sustainable resources is mainly due to the materials is derived from natural sources that are abundantly available. Their use would also contribute to global sustainability without depletion of limited resources. Also, sustainable resources are comparatively easy to handle with no or less toxicity and health related issues.[12,13] The increasing number of research studies devoted to the development of renewable resources based resins such as epoxy, polyester, polyurethane, Polyesteramide; reveals the great potential of renewable raw molecules and their ability to substitute for petrochemical-based materials.[14–20] In this context, many kinds of raw materials from renewable resources such as plant oils, rosins, terpenes, and sugars have been investigated as building blocks of polymeric materials.[21,22]

Itaconic acid (IA) as a renewable material is attracting a lot of interest.[23] It is a white crystalline unsaturated dicarbonic acid. IA produced by the fermentation of Aspergillus terreus from sugar is a efficient and promising diacid containing an active 11-disubstituted vinyl group.[23–25] The Aspergillus terreus is the most frequently used commercial producer of IA, several attempts also been made to find other microorganisms that are suited for efficient production of IA. Among the filamentous fungi, some ustilaginales species are known to produce IA. Because of its green synthetic procedure and great potential as a sustainable platform chemical and a building block for the production of polymers.[26] Former studies using IA as a starting material for polymeric materials mainly focused on two directions. One is the radical copolymerization of itaconate esters to prepare vinyl copolymers, and the other is the condensation polymerization of IA with diols to form polyesters.[27,28] The IA possessing two carboxyl groups and one carbon–carbon double bond, which has been proved to be suitable for the synthesis various resin for coating application. IA has also been reported as a potential raw material for synthesis various resin such as epoxy,[29] polyesteramine,[30] polyester,[27–31] polyamide.[32] A variety of biorenewable raw materials (such as vegetable oils, polysaccharides, natural rubbers, and their derivatives) is used for producing UV-curable coatings. Therefore, the combination of renewable resource and UV-curing method will provide us a ‘green+green’ strategy to prepare the thermosetting resins derived from biomass, which should have a bright future.

The aim of the present work was to utilize the IA to develop the UV curable PUA coating. The synthesis of IA based UV curable PUA resin was carried out in the two steps. The first step was the conversion of carboxylic group of itaconic acid in to the hydroxyl functional polyol via condensation reaction with 16 hexanediol in presence of pTSA catalyst. In second step, IA based polyol and excess IPDI react to prepare the NCO terminated PU prepolymer. Subsequently, NCO terminated PU prepolymer react with HEMA to incorporate the unsaturation at the end group. The developed resin was characterized for their physicochemical and FTIR, ^1^H and ^13^C NMR spectroscopic analysis for structural elucidation. The synthesized UV curable IA based PUA resin incorporated in various concentrations into the conventional PUA. The effects of varying concentration of synthesized UV curable PUA resin on rheology, crystallinity, thermal and coating properties were evaluated. The synthesized UV curable PUA coatings showed interesting mechanical, chemical, solvent and thermal properties as compared to the conventional PUA.

## 2. Experimental

### 2.1. Materials

Itaconic acid [IA], 16-hexanediol, 4-methoxyphenol and para-Toluene sulphonic acid, 2-hydroxyethyl methacrylate and acetone were procured from SD Fine Chemicals Ltd. Mumbai, India. Isophorone diisocyanate was received from Bayer Materials Science Ltd., Mumbai, India. Dibutyltin dilaurate (DBTDL, as the catalyst) was purchased from Amrut Industries Ltd Mumbai, India. Photoinitiator 2-Hydroxy-1-{4-[4-(2-hydroxy-2-methyl-propionyl)-benzyl]-phenyl}-2-methyl-propan-1-one (IRGACURE® 127) was received from BASF, India. Dipropylene glycol diacrylate (Photomer® 4226) as reactive diluent was received from shepherd color company India.

### 2.2. Synthesis of itaconic acid based polyol (IAP)

IA (17.62 g, 135.4 mmol) and 16-hexanediol (32.01 g, 270.8 mmol) were charged into a round bottom four-necked flask equipped with a mechanical stirrer, a thermometer, a reflux condenser and a nitrogen inlet. The PTSA (0.5 mol% relative to itaconic acid) as the catalyst and MEHQ (0.5 wt% relative to the total weight of itaconic acid and 16 hexanediol) as the free radical polymerization inhibitor was added into the reaction flask. The reaction mixture was stirred and temperature was increased gradually upto 140 °C in presence of N_2_ atmosphere and progress of reaction was monitor by determining the acid value at regular interval of time. The reaction was stopped when acid value of reaction mixture reached below 10 mg of KOH per gram of resin. The synthesized IA based polyol was characterized for hydroxyl value, FTIR, ^1^H and ^13^C NMR analysis.

### 2.3. Synthesis of UV curable polyurethane acrylate resin

In the second step, 15.83 g (46.90 mmol) IA based polyol along with acetone (15 ml) were charged into the round bottom flask under stirring in presence of N_2_ gas inlet. After formation of homogenous mixture 0.1 wt% DBTDL and 21.93 g (98.67 mmol) IPDI mixed in acetone (10 ml) added dropwise under constant mechanical stirring. The reaction temperature was increased slowly up to 65 °C and maintained for next 3 h. The progress of reaction was monitored by FTIR analysis. The reaction was continued until the hydroxyl functional groups were completely consumed, it was confirmed by disappearance of the −OH peak from FTIR spectrum. Afterwards, 12.12 g HEMA (93.98 mmol) was added into the round bottom flask. The reaction temperature was maintained at 65 °C for next 4 h. The progress of reaction was monitor by FTIR analysis. The reaction was continued until peak corresponding to NCO functional group completely disappeared from FTIR spectrum. The reaction mixture was cool to room temperature and IA based UV curable PUA resin characterized for FTIR, ^1^H and ^13^C NMR analysis.

### 2.4. Formulation of UV curable PUA coating

The synthesized IA based PUA resin incorporated in various concentrations into the conventional PUA resin as shown in Table 1. The reactive diluent and photo initiator concentration kept constant in all formulations. The formulated coating was applied over the mild steel (MS) panels using bar applicator at 60-μm wet film thickness. The panels were cured by UV curing machine (UNIQUE UV Curing Machine, India) with the high-pressure mercury lamp (UV curing lamp Power 4 kW, wavelength: 30–400 nm) the exposure time was 20 s. The UV cured coatings were characterized for mechanical, chemical resistance, solvent resistance and thermal properties.

**Table 1.** Formulation of UV curable PUA coatings.

Code	PUA	IA based PUA	Reactive diluents	Photo initiator
PUA	76	0	20	4
PUA 1	56	20	20	4
PUA 2	36	40	20	4
PUA 3	16	60	20	4
PUA 4	0	76	20	4

### 3. Characterization

### 3.1. Physico-chemical analysis of resin

The acid value of IA based polyol was determined by the volumetric titration method according to ASTM D1980-87. The known quantity of resin sample dissolved in neutral butanol-toluene mixture and 3–4 drop of phenolphthalein indicator added, swirling gently and titrated against the 0.1 N alcoholic KOH solution. Equation (1) was used to determine the acid value of IA based polyol.

(1) $$\text{Acid value}=\frac{B\;\times N\times \;56.1}{W}$$

where *B* = Burette reading (ml), *N* = Normality of alcoholic KOH solution, *W* = weight of sample (g).

The hydroxyl value of IA based polyol was determined by acetic anhydride-pyridine method according to ASTM D4274-16. The calculation of hydroxyl value was done by using Equation (2).

(2) $$\text{Hydroxyl value}=\;\frac{\left[B-S\right]\times \;N\times \;56.1}{W}$$

where *B* = Burette reading for blank (ml), *S* = Burette reading for sample (ml), *N* = Normality of alcoholic KOH solution, *W* = weight of sample (g).

### 3.2. Instrumentation

#### 3.2.1. FTIR analysis

The chemical structure of the IA based polyol, NCO-terminated PU prepolymer and PUA resin were identified by FTIR on a Bruker ATR spectrophotometer, USA. The spectra were observed in the 600 – 4000 cm^−1^ wavelength range.

## ^1^H and ^13^C nuclear magnetic resonance spectroscopy

The supportive confirmation of chemical structure was given by ^1^H and ^13^C nuclear magnetic resonance (NMR) spectroscopy. ^1^H and ^13^C NMR spectra of the product were analyzed using Bruker DPX 800 MHz spectrophotometer with dimethyl sulphoxide (DMSO) as solvent.

### Rheology

A rheometer (MCR 101, Anton Paar, Austria) with a parallel plat assembly was used to investigate the rheological behavior of the resins. Cone and plate rheometer (diameter: 25 mm, D-CP35-SN0) were separated by a distance of 0.5 mm during the rheological analysis. The rheological data analysis was performed using Rheoplus/32 V3.40 software supplied by the manufacturer. A temperature of 30 ± 0.5 °C was maintained constant during the rheological measurement.

### DSC analysis

The glass transition temperature (*T*_g_) of cured films was estimated using differential scanning calorimetry (DSC) TA Q100 analyzer (T. A. Instrument, USA). The film sample weighed accurately in aluminum pan and heated in 40 – 120 °C temperature range with heating rate 10° C/min under nitrogen.

### Thermogravimetric analysis

Thermogravimetric analysis (TGA) of cured films was conducted on Perkin Elmer TGA 4000 instrument under nitrogen and air atmosphere. Thermal analysis monitored in the temperature range 40–700 °C with 20 °C/min heating rate.

### XRD analysis

Wide-angle X-ray diffraction (XRD) was used to study the interplanar distances (d-spacing) in the PU crystal line samples XRD patterns were collected with a Rigaku RotaFlex operating with CuKa radiation, 50 kV, 100 mA, and equipped with a graphite monochromator. The data collection was recorded in the range of 2*θ* = 5–50° with a step of 0.02° and 2 s/step.

### 3.3. Coating characterization

Coatings were evaluated for mechanical (Adhesion, Hardness, Flexibility and Impact resistance properties), chemical (acid, alkali), solvent scrub resistance, water absorption and gel content as per ASTM standard.

#### Mechanical properties

Coating adhesion to metal substrate was examined by cross-cut test according to ASTM D-3359. In cross-cut test, a lattice marking of 1 cm^2^ on coating was made till the metal surface was exposed and adhesive tape was then applied over the lattice marking. The adhesive tapes pull out and lattice marking of coating was examined for adhesion failure as per the ASTM standard. The conical mandrel tester was used to determine the flexibility according the ASTM D-522 and the capacity of a coating material to survive a high force in a short period is studied by impact resistance tester (ASTM D-2794). The impact resistance test was carried out through dropping a 2 lb weight ball from maximum height of 60 cm and weight dropped onto the coated surface (forward impact) and on the reverse of the panel with reference to the coated surface (reverse impact). Scratch hardness (IS-104) of the coating was measured on hardness tester. The MS panel was fixed onto a movable base with the coated surface facing upward. A needle rest on the coating and moved, if the needle makes contact with the metal substrate then the coating is said to have failed at that point for that weight. Pencil hardness was also used to study the hardness of coating according to the ASTM D-3363. The 6B to 6H range of pencils were used for the test.

#### Chemical resistance

Chemical resistance property of the coatings performed according to the ASTM D-1308. The coated panel immersed in 5% w/w aqueous sodium hydroxide (NaOH) solution and 5% v/w hydrochloric acid (HCl) solution separately for 24  h at ambient temperature and its edges were sealed with adhesive tape. The coated panel was monitored for any visible defects like softening of film, blistering and separation of film from substrate.

#### Solvent scrub resistance

Solvent scrub resistance (ASTM D-4752) of coating was evaluated by using methyl ethyl ketone (MEK). Solvent scrub resistance of cured coatings was studied by rubbing the coating with a piece of white cotton saturated with solvent and maximum rubbing cycles were 200.

#### Gel content

Gel content of the cured coatings was determined by putting a known weight of coating sample (Wi) into xylene: DMF solvent (50: 50 by volume) for 24 h. Next day, a coating was removed from solvent and the residue dried in oven to achieve constant weight (Wr). Gel content calculated by using the Equation (3).

(3) $$\text{Gel content}\;=\;\frac{W_{\text{w}}}{W_{\text{d}}}\times \;100$$

where *W*_w_ = wet coating weight, *W*_d_ = dry coating weight

#### Water absorption

Water absorption of cured coatings was measured according to ASTM D-570. The cured coating was dried in oven until a constant weight achieved (*W*_1_), then dipped into water at room temperature for 24 h. The coating film was removed from the water and dried with paper towel and weighed (*W*_2_). Water absorption was calculated from the difference in the weights of the sample before and after soaking the water according to Equation (4).

(4) $$\text{Water absorption}\;\left(\%\right)=\frac{\left(W_{f}-W_{i}\right)}{W_{i}}\;\times \;100$$

where, *W*_1_ = weight of the sample after immersion in water.

*W*_2_ = weight of the sample before immersion in water.

## 4. Result and discussion

### Physicochemical analysis

UV curable PUA resin was synthesized from polyol based on sustainable resource originated from IA, IPDI and HEMA. UV curable PUA resin synthesis was carried out in the two steps. The progress of IA based polyol synthesis reaction was monitored by physicochemical analysis. Physicochemical analysis of IA based polyol is shown in Table 2. In second step, IA based polyol and excess IPDI react to prepare the NCO terminated PU prepolymer. Subsequently, NCO terminated PU prepolymer react with HEMA to incorporate the unsaturation at the end group of PUA resin.

**Table 2.** Physicochemical analysis of IA based polyol.

Sample	Acid Value (mg of KOH/g resin)	Hydroxyl value (mg of KOH/g resin)
IA based polyol	8.24	166.54

### FTIR analysis

**Figure 1.**
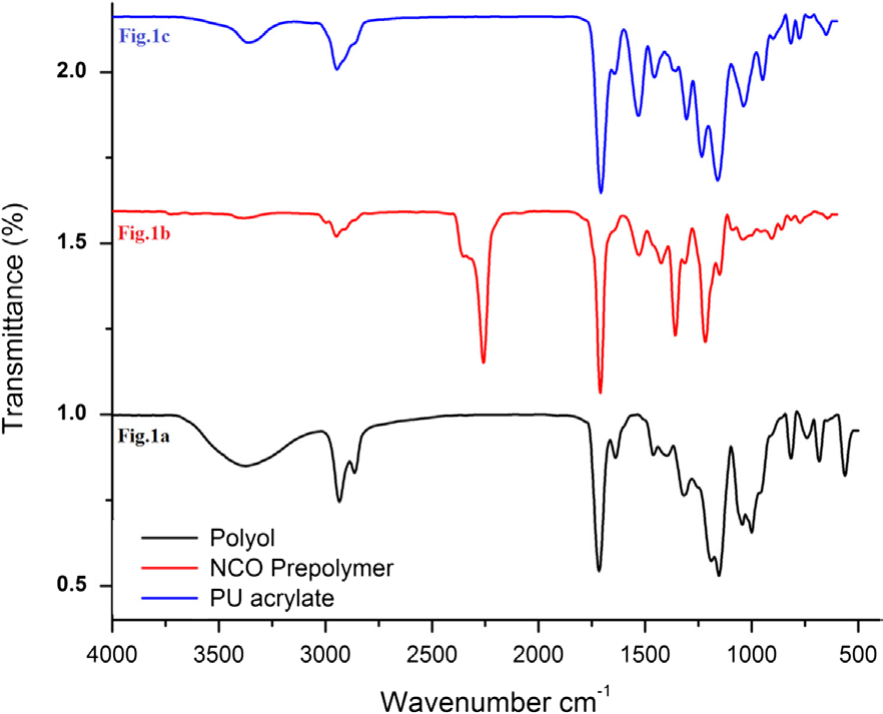
FTIR analysis of (a) itaconic acid based polyol (b) NCO terminated PU prepolymer (c) PUA resin.

The FTIR spectra of IA based polyol, NCO terminated PU prepolymer and PUA resin is given in Figure 1. The Figure 1(a) displays FTIR spectrum for IA based polyol. The peaks at 925 and 1090 cm^−1^ are attributed to the unsaturation (C=C) and ether (C–O–C) linkage. The IA based polyol spectrum has strong absorption peak at 1716 cm^−1^ is assigned to the C=O stretching of ester. Asymmetrical and symmetrical stretching of CH_2_ and CH_3_ appeared at 2926 and 2866 cm^−1^ respectively. The broad absorption band at 3374 cm^−1^ corresponds to the −OH groups, suggesting that the reaction of IA and 16-hexanediol has taken place to synthesize the IA based polyol. The FTIR spectrum in Figure 1(b) show spectrum of NCO terminated PU prepolymer. In the FTIR spectrum of NCO terminated PU prepolymer peak assigned to 3383 and 1424 cm^−1^ for the stretching vibration of N–H and C–N respectively, which are caused by the reaction of NCO groups of IPDI and −OH groups of polyol.[31,33] The prepolymer spectrum show strong absorption peak at 1716 cm^−1^ viz. assigned to the C=O stretching of ester. The characteristic absorption peak of NCO terminated PU prepolymer at 2258 cm^−1^ belongs to the stretching vibration of the unreacted NCO functional group. The FTIR spectrum in Figure 1(c) is for IA based UV curable PUA resin. The FTIR spectrum of PUA resin does not show the characteristic peak of –NCO at 2258 cm^−1^. It indicates that all NCO groups react with the hydroxyl groups of HEMA and PUA resin is synthesized. The absorption peak at 810 cm^−1^ illustrates that the acrylate C=C bond has been successfully incorporated at the end of PU acrylate resin chains. From above FTIR results, we conclude that IA based UV curable PUA resin is successfully synthesized.

## ^1^H and ^13^C NMR analysis

**Figure 2.**
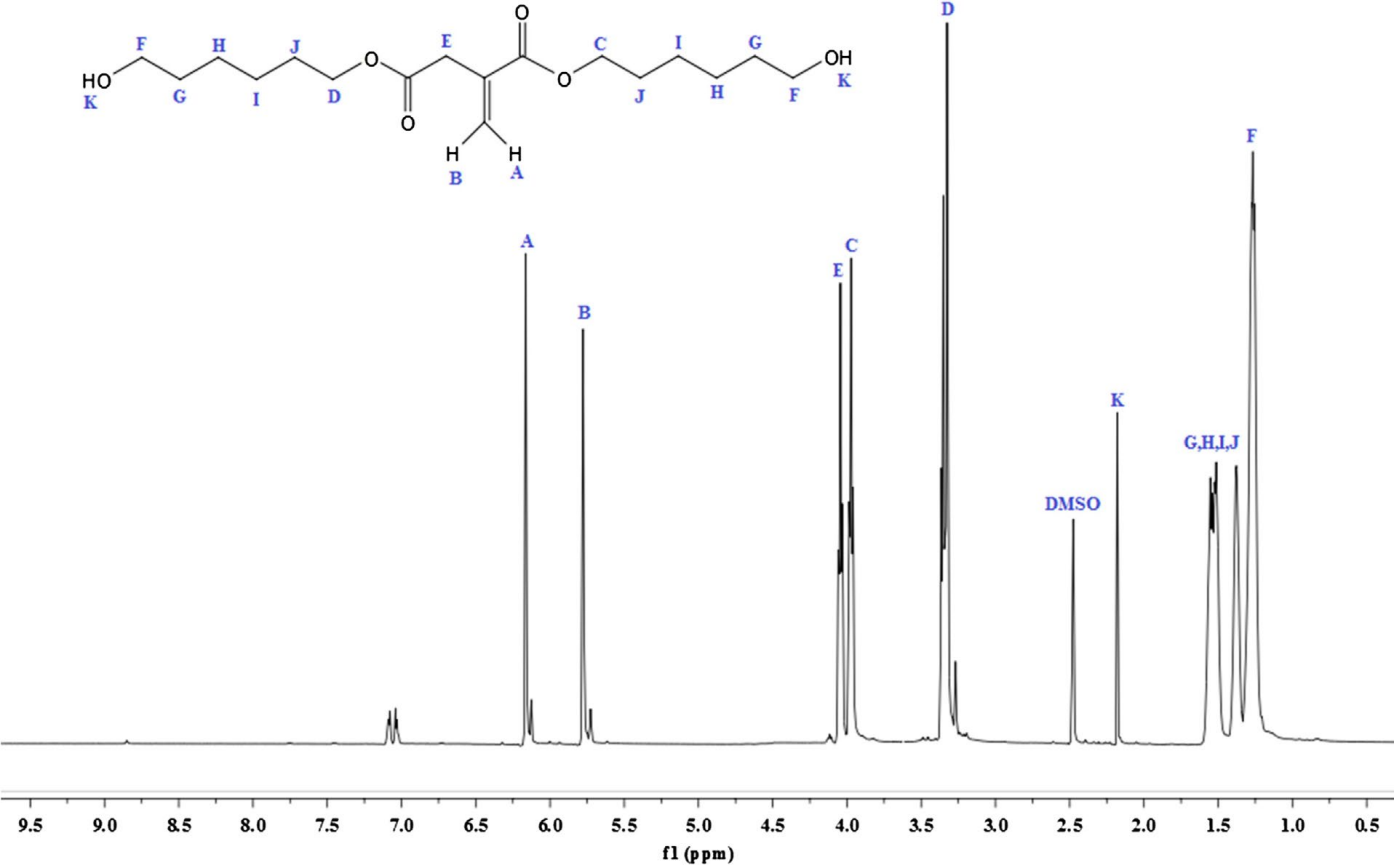
^1^H-NMR analysis of IA based polyol.

**Figure 3.**
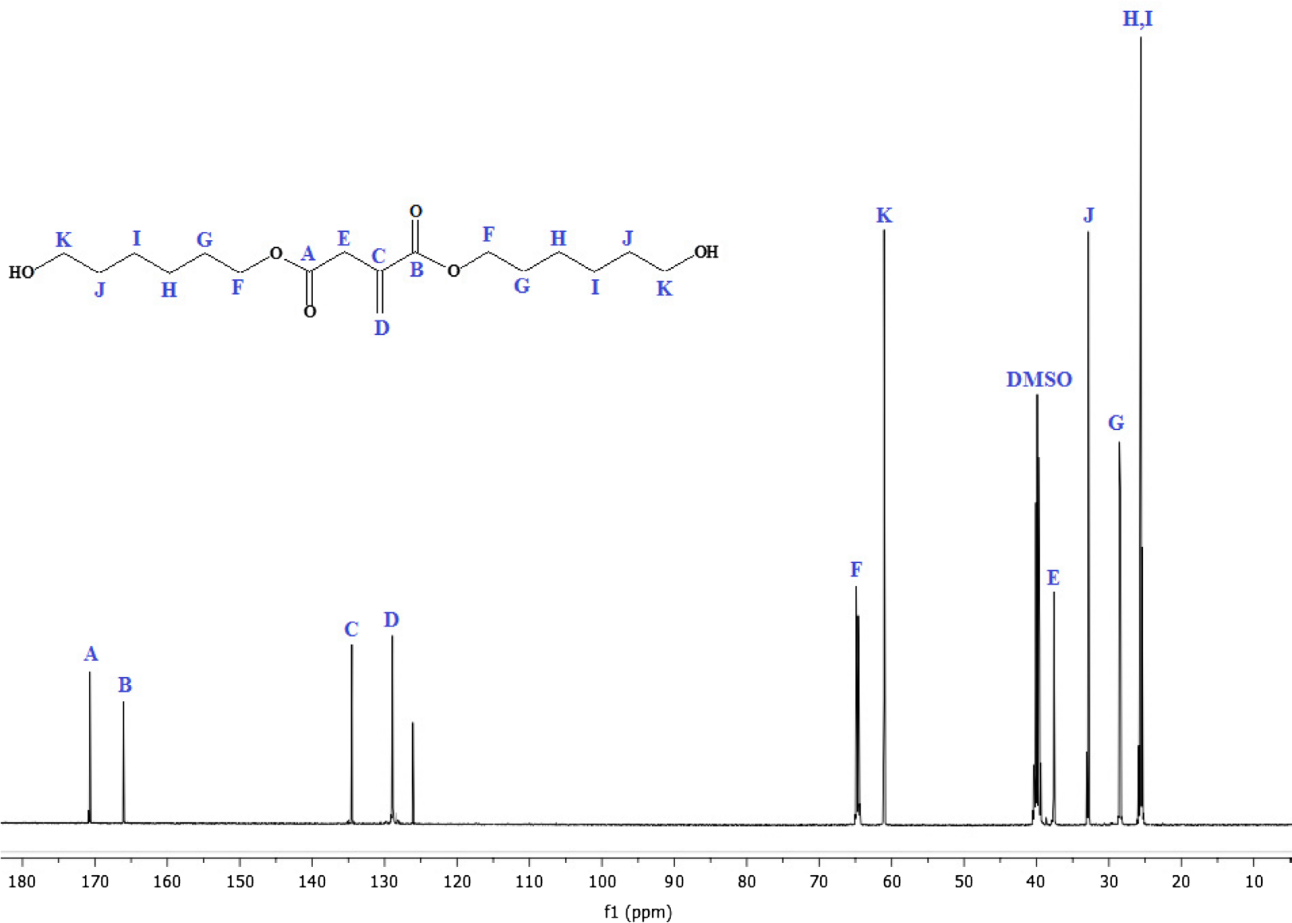
^13^C-NMR analysis of IA based polyol.

**Figure 4.**
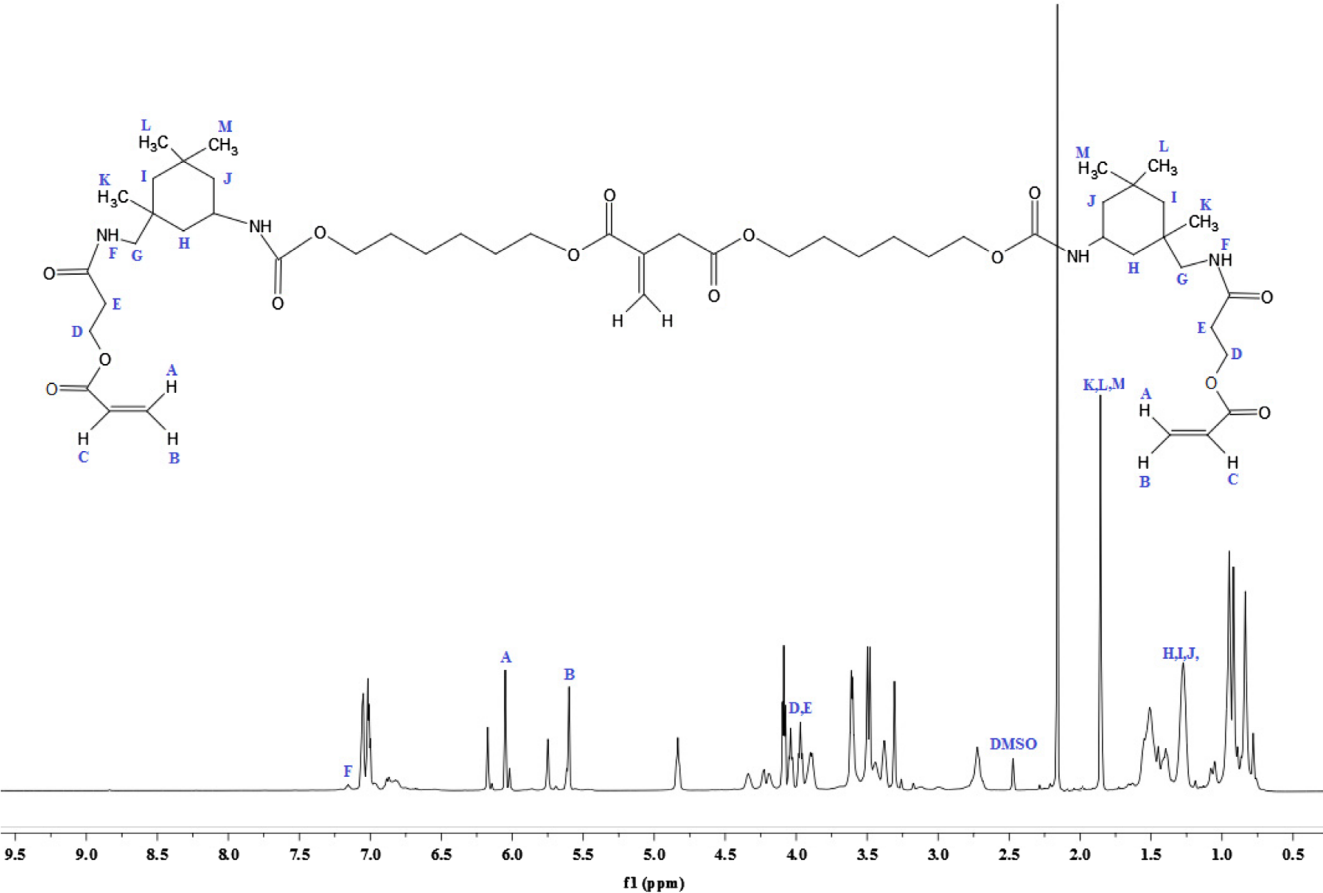
^1^H-NMR analysis IA based PU acrylate.

**Figure 5.**
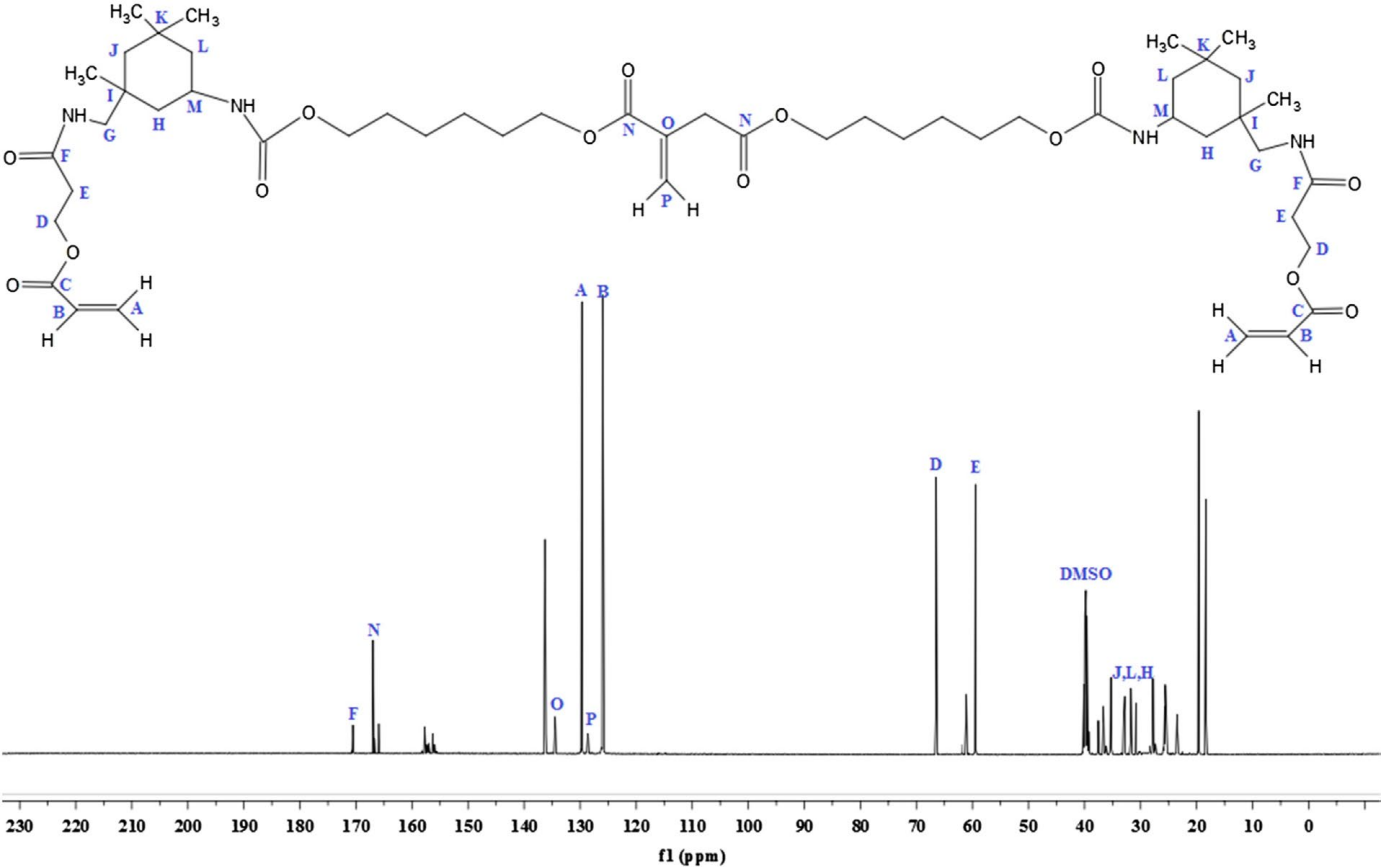
^13^C-NMR analysis IA based PU acrylate.

^1^H-NMR analysis of IA based polyol is shown in Figure 2. The signal obtained as multiplet at 1.33 and 1.58 ppm (G, H, I, J, 8H) could be attributed to the methylene protons of aliphatic chain. The sharp singlet at 2.18 ppm (k, 1H) corresponds to the proton of hydroxyl group; the absorption shift towards the downfield is due to the electronegative effect of oxygen atom. The multiplet signal at 3.96 ppm (C, 2H) and 3.34 ppm (D, 2H) correspond to the proton adjacent to carbonyl group of acid. The peak shift towards the downfield due to the de-shielding caused due to electronegative effect of carbonyl group. The multiplet was assigned at 4.04 ppm (E, 2H) for the proton associating with a carbon atom near to the carbonyl group. The singlet corresponds to the vicinal bond of IA resonates at 6.16 ppm (A, 1H) and 5.77 ppm (B, 1H) that deshielded due to the movement of the electrons in the pi bond of the carbon–carbon double bond.[31] The ^13^C-NMR analysis of IA based polyol show in Figure 3. The two sharp peaks appear at 25 ppm (H, I) and 33 ppm (J) nearest to the TMS correspond to the carbon atom of aliphatic chain. There is absorption at 62 ppm (K) for the carbon of CH_2_ that deshielded due to the adjacent hydroxyl group. There is absorption at 65 ppm (F) correspond to the carbon near to carbonyl group and shift towards down field in spectrum due to deshielded effect of carbonyl group. The peaks at 128 ppm (D) and 135 ppm (C) correspond to the double bonded carbon atom. The characteristic absorption peak that appears at extreme end of spectrum at 172 (A) and 166 ppm (B) corresponds to the carbonyl carbon of acid and ester respectively. Both of the carbon atoms appear at nearly same chemical shift value and they are highly deshielded. Thus from the ^1^H and ^13^C NMR spectrum data, we can confirm that IA based polyol resin is successfully synthesized. The ^1^H-NMR spectrum of IA based PUA resin is shown in Figure 4. The peak at 1.28 ppm is assigned to the cycloaliphatic protons. The triplet peaks at 3.95 ppm (E, 2H) and 4.10 (D, 2H) ppm are assigned for the CH_2_ protons. The peak attributed at 6.04 ppm (A, 2H) and 5.60 ppm (B, 2H) are assigned to the protons of acrylate (CH=CH_2_) which is the end group of PUA resin. The peak attribution of protons at the down field is due to the movement of the electrons in the pi bond of the carbon–carbon double bond.[34] The ^1^H-NMR spectrum show characteristic peak at 7.15 ppm (F, H) which is very broad and weak without any distinct coupling to the hydrogen on an adjacent carbon atom corresponds to the hydrogen attached to nitrogen. All other absorptions are nearly same as explained above ^1^H-NMR section of IA based polyol. ^13^C-NMR analysis of IA based PU acrylate resin is shown in Figure 5. The signals at 66.48 ppm (D) and 59.45 ppm (E) are assigned for the carbon of CH_2_–CH_2_ linkages. The signals in 30–35 ppm region are assigned for the carbon of cycloaliphatic ring. The signals of 129.75 ppm (A) and 126.01 ppm (B) are assigned for the carbon of acrylate (CH=CH_2_). The peaks of ester carbonyl carbon and urethane-carbonyl carbon signal appear at 166.95 ppm (N) and 170.5 ppm (F) respectively. The IA characteristic double bonded carbon are assigned to signal at 134.5 ppm (O) and 128.6 ppm (P). All other absorption appeared are nearly same as explained above. Thus from the ^1^H and ^13^C NMR spectrum data, it may be concluded that IA based polyol is successfully utilized for the synthesis of UV curable PUA resin.

### Rheology

**Figure 6.**
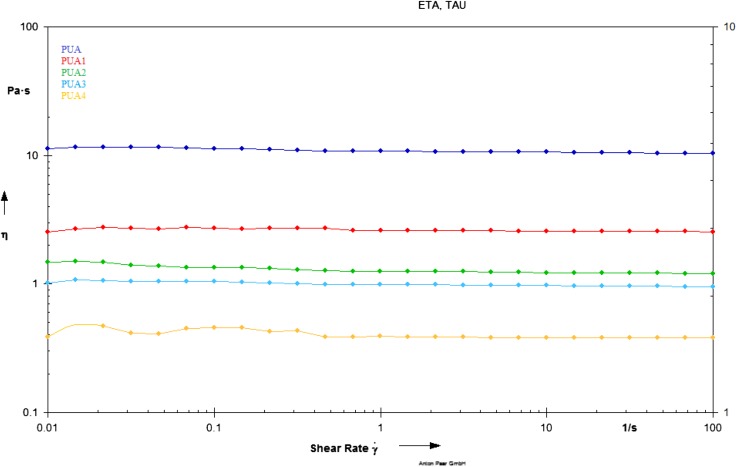
Rheology study of PUA, PUA1, PUA 2, PUA 3 and PUA 4 resins.

The viscosity of resin is one of the most important property and it is key factor to determine the end application of thermosetting resin. The lower viscosity of resin will undoubtedly play a positive role in improving its overall performance as final coatings. The chain structure and molecular structure of a polymer determine its rheological properties.[35] The rheological behavior of conventional PUA, PUA1, PUA2, PUA3 and PUA resins at variable shear rate is studied and results are in Figure 6. From Figure 6, increase shear rate does not show any change in the viscosity of conventional PUA, PUA1, PUA2, PUA3 and PUA resins. In addition, viscosity of PUA4 resin is found to be lowest as compared to the conventional PUA, PUA1, PUA2 and PUA3 resin. From above study, we conclude that viscosity of conventional PUA resin drop dawn, as IA based PUA resin concentrations increase.

## 5. Coating characterization

### DSC analysis

**Figure 7.**
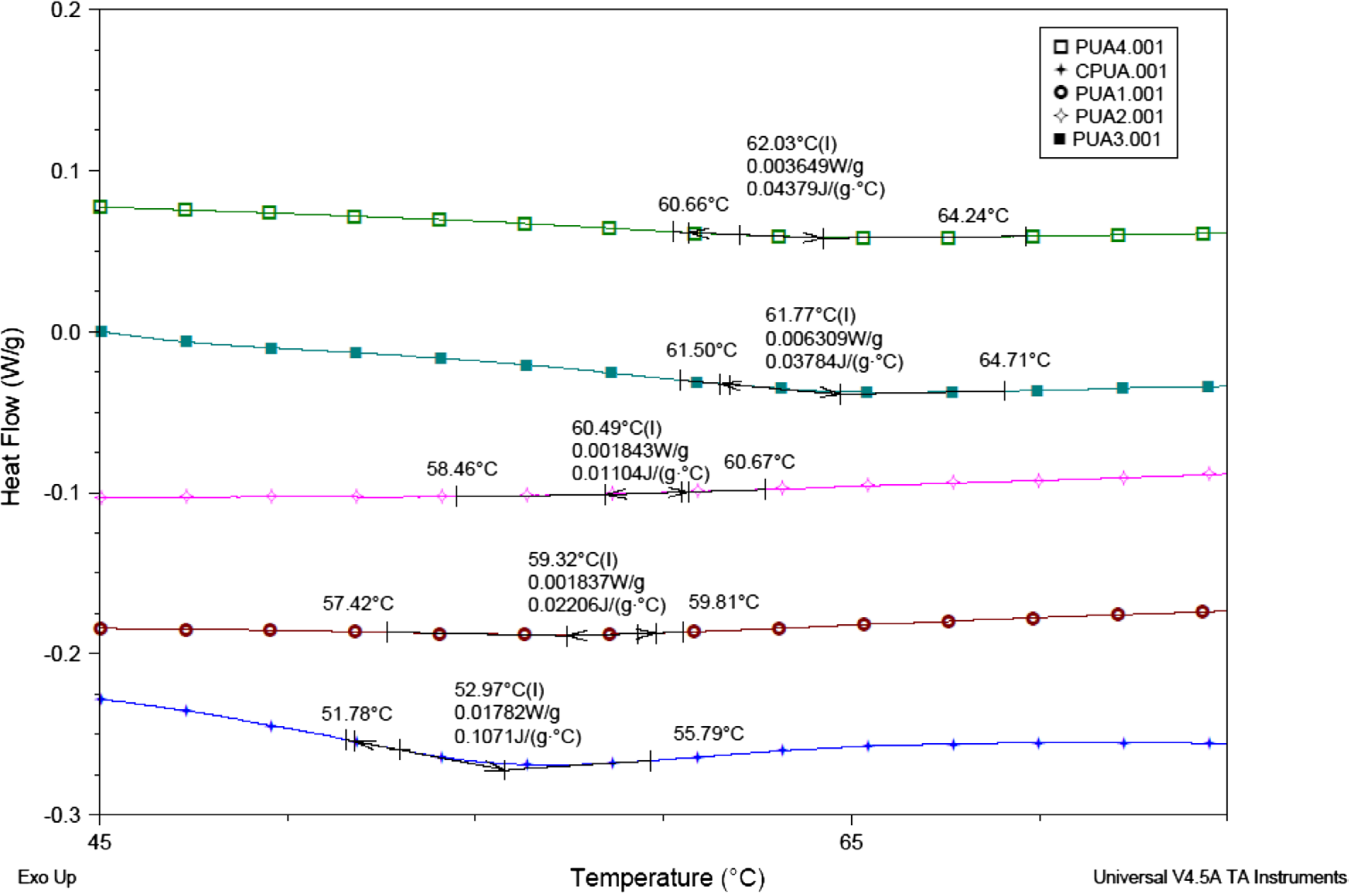
DSC analysis of PUA, PUA1, PUA 2, PUA 3 and PUA 4 coatings.

The *T*_g_ of UV cured coatings was determined by DSC analysis and it is shown in Figure 7. The DSC analysis shows the *T*_g_ (midpoint) of PUA, PVA1, PUA2, PUA3 and PUA4 values as 52.97, 59.32, 60.49, 61.77 and 62.03 °C respectively. The dense cross-linked structures have higher value of *T*_g_ than less cross-linked cured coating. The crosslink density depends on the functional groups present in molecules and more the functional groups present in the resin backbone the higher the cross-linked structure.[36,37] IA based PUA resin has one extra reactive site in the backbone for UV curing as compared to the conventional PUA resin. Therefore, UV cured IA based PUA coatings have higher crosslink density compared with the conventional PUA cured coating. In addition, the resulting urethane linkages also contribute to compact network through intermolecular hydrogen bonding which would also reduce the chain mobility of final structure and increase the *T*_g_ significantly. Hence, it is observed that *T*_g_ of the UV cured PUA coatings shifted to the higher value with the IA based PUA concentration increased into the coating. It may be due to the IA based PUA coatings formed dense cross linked structure than conventional PUA coating.

### TGA analysis

**Figure 8.**
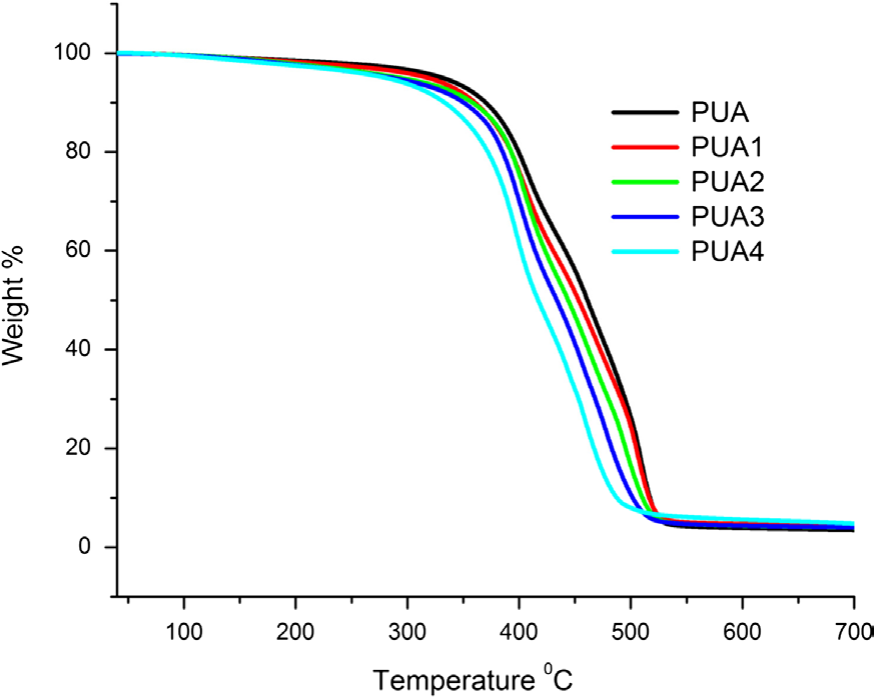
TGA analysis of PUA, PUA1, PUA 2, PUA 3 and PUA 4 coatings.

Thermogravimetric analysis (TGA) method was used to study the changes in physical and chemical properties of materials as a function of increasing temperature. TGA analysis of cured coating is shown in Figure 8. Thermal degradation of PU is a very complex mechanism due to variety of products formed in the process that are indicative of the complexity of the degradation process. Thermal stability of UV cured PUA coatings mainly depends upon the number of hard and soft segments present in the final structure. The urethane linkages (–NH–COO) and structure of isocyanate used for curing are responsible for the hard segment while long alkyl chains of polyols presents soft segments in the final cured structure.[38] The values of 5 wt% (*T*d_5%_), 30 wt% (*T*d_30%_) loss degradation temperature and residual weight% (char yield) at 700 °C are listed in Table 3. As shown in Figure 8, *T*d_5%_ temperature of the cured coatings decreased from 331.67 °C for PUA to 280.33 °C for PUA4.

**Table 3.** Thermo gravimetric analysis of PUA, PUA1, PUA2, PUA3 and PUA4 coatings.

Coatings	5% (°C)	30% (°C)	Ts (°C)	Residues at 700 °C
PUA	331.67	418	204.82	3.51
PUA1	318	410	200.90	3.35
PUA2	294	407	199.43	3.99
PUA3	287	400	196.00	4.02
PUA4	280.33	389	190.61	4.78

Thermal stability of the UV cured PUA coatings are characterized by the statistic heat-resistant index temperature (*T*s). The temperatures at 5% (*T*d5) and 30% weight loss (*T*d30) of the cured coating obtained in thermogravimetric analysis is used to calculate the *T*s (Equation (5)). The calculated value of statistic heat-resistant index (*T*_s_) of cured IA based PUA coatings have lower value than cured PUA coating.

(5) $$T_{\text{s}}=0.49\;\left[Td5+0.6\;\left(Td30-Td5\right)\right]$$

where *T*_s_ = statistic heat-resistant index temperature

The char yield value at 700 °C for PUA, PUA1, PUA2, PUA3 and PUA4 coatings are 3.51, 3.35, 3.99, 4.02 and 4.78 respectively. The coating degradation temperature not only has a relationship with the chemical structure of the network, it also has close ties with crosslink density of the network. The cross-linked density of coating increases with functional group and high cross-linked network has high thermal stability.[39] IA based PUA resin have more unsaturation in the backbone as compared to the conventional PUA resin and form more crosslinked structure when passed through the UV lamp. This higher cross linking density of IA based PU coating may be enhancing the thermal resistance properties. From TGA analysis study revealed that as the IA based PUA resin concentration increased in PUA coatings, thermal stability increase.

### XRD analysis

**Figure 9.**
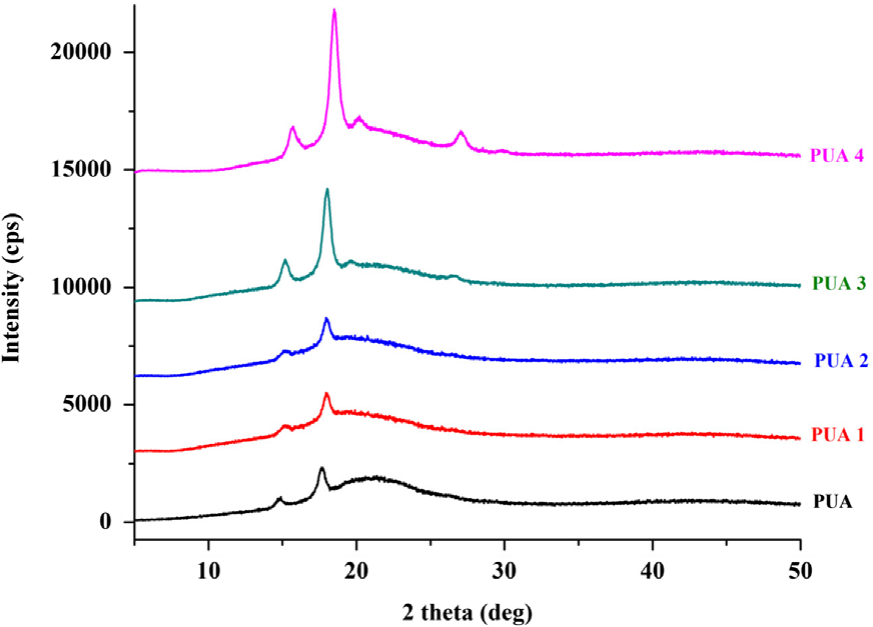
XRD patterns of PUA, PUA1, PUA2, PUA3 and PUA4 coatings.

The degree of crystallinity of the coatings was determined from XRD patterns using the PFM program. The 2*θ* scan range was 5–50°, because all the intense crystalline peaks are found in this range. The X-ray pattern of the UV curable PU coatings is shown in Figure 9. The XRD patterns of PUA coating exhibits broad peaks at about 2*θ* = 15° and 18°, indicating some degree of crystallinity. These peaks are assigned to the scattering from PUA chains with regular interplanar spacing. The peaks intensity at about 2*θ* = 15° and 18° is increase as the mass proportion of IA based PUA resin increases. The determined percent crystallinity of PUA, PUA1, PUA2, PUA3 and PUA4 coatings from XRD analysis is 3.44, 3.62, 4.15, 5.24 and 6.79 respectively (Table 4). IA based PUA resin has one extra reactive site in the backbone for UV curing as compared to the conventional PUA resin and hence, higher crosslink structure formed compared to the conventional PUA coating.[40,41] Thus, as concentration of IA based PUA resin increase into the coatings, the coating becoming more rigid and crystalline.

**Table 4.** Percent crystallinity of PUA, PUA1, PUA2, PUA3 and PUA4 coatings.

Coatings	Crystallinity (%)
PUA	3.4475
PUA1	3.6229
PUA2	4.1554
PUA3	5.2454
PUA4	6.7993

### Gel content

**Figure 10.**
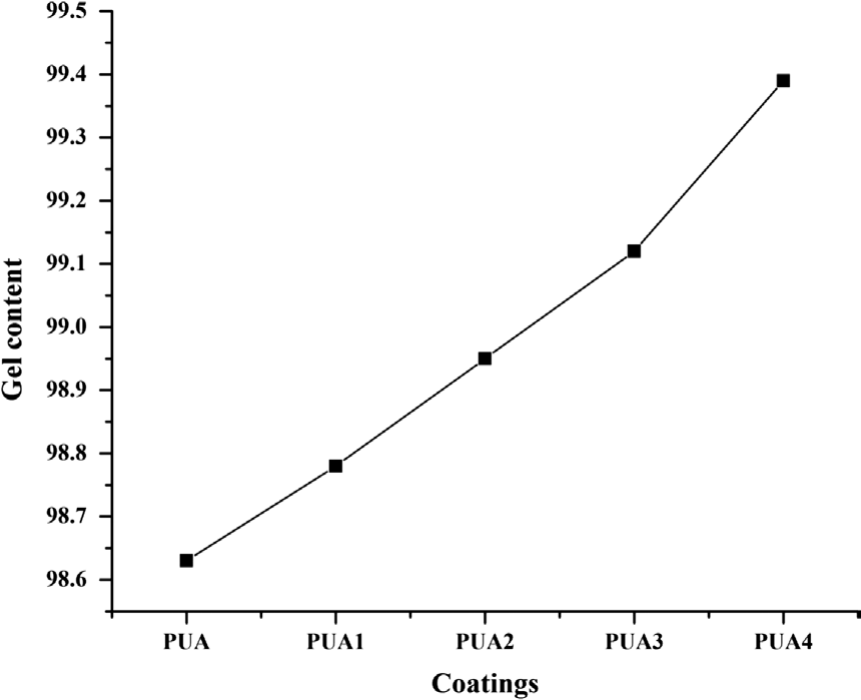
Gel content of PUA, PUA1, PUA 2, PUA 3 and PUA 4 coatings.

The gel content of UV cured coatings was evaluated in methyl ethyl ketone (MEK) solvent for 24  h and result shown in Figure 10. The gel contents of PUA, PUA1, PUA2, PUA3 and PUA4 coatings are 98.63, 98.78, 98.95, 99.12 and 99.39 respectively. The results show that gel content of UV cured coating increase as the concentration of IA based PUA resin increase. The gel content is correlated to the crosslinked structure of cured coating film.[41] The cross-linked density of coating is related to the number of reactive sites present in the resin structure and more the reactive sites higher is the cross-linking density. IA based PUA resin have more reactive sites in the structure as compared to the conventional PUA resin. Therefore IA based PUA coatings formed have more crosslinked structure when cured through the UV irradiation lamp. The penetration of solvents in UV curable IA based PUA coating become difficult. All the coatings show gel content over 98% and confirm that coatings have excellent curing.

### Water absorption

**Figure 11.**
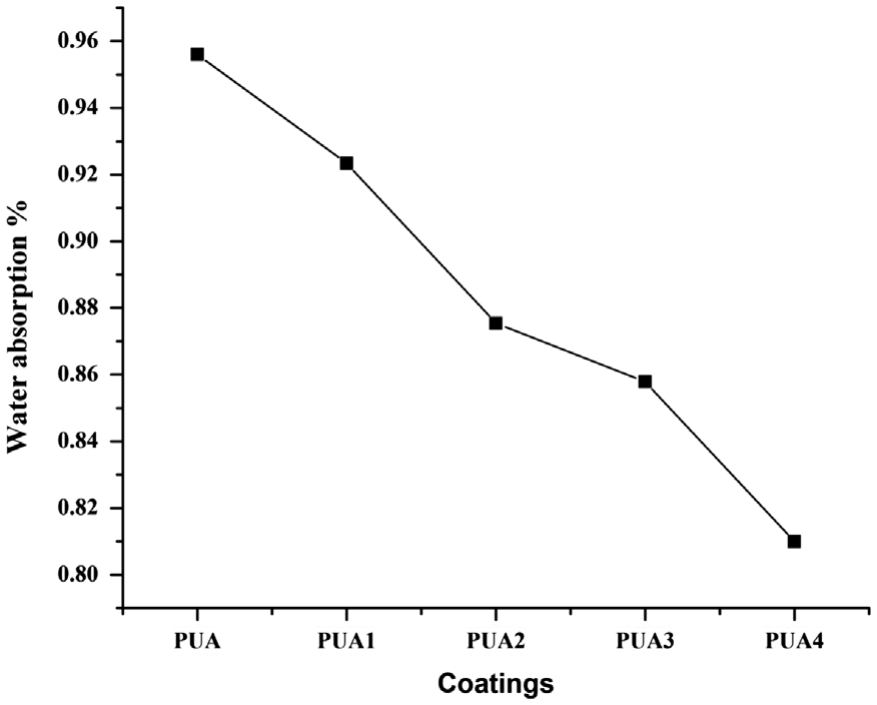
Water absorption test of PUA, PUA1, PUA 2, PUA 3 and PUA 4 coatings.

**Scheme 1.**
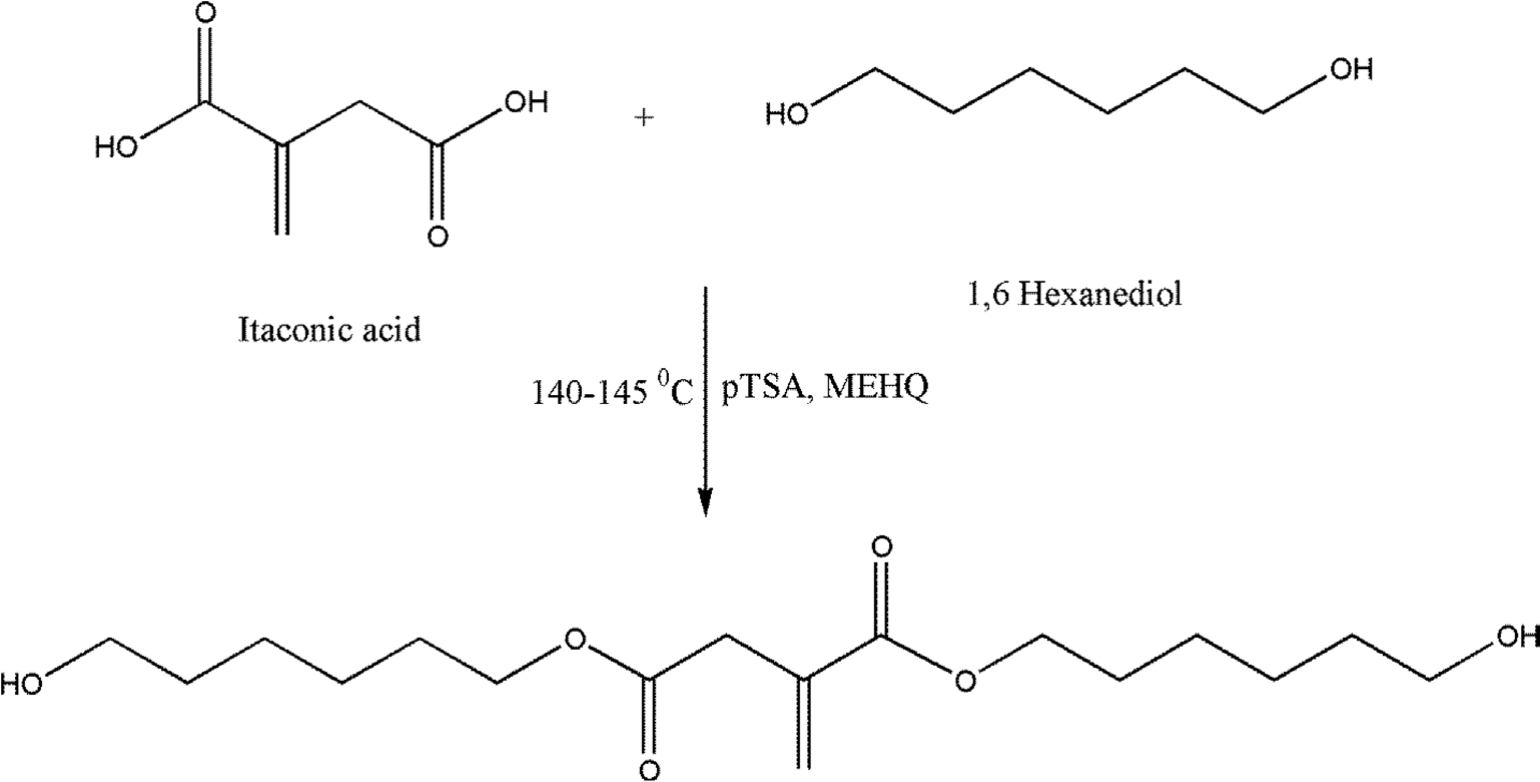
Synthesis route for IA based polyol.

**Scheme 2.**
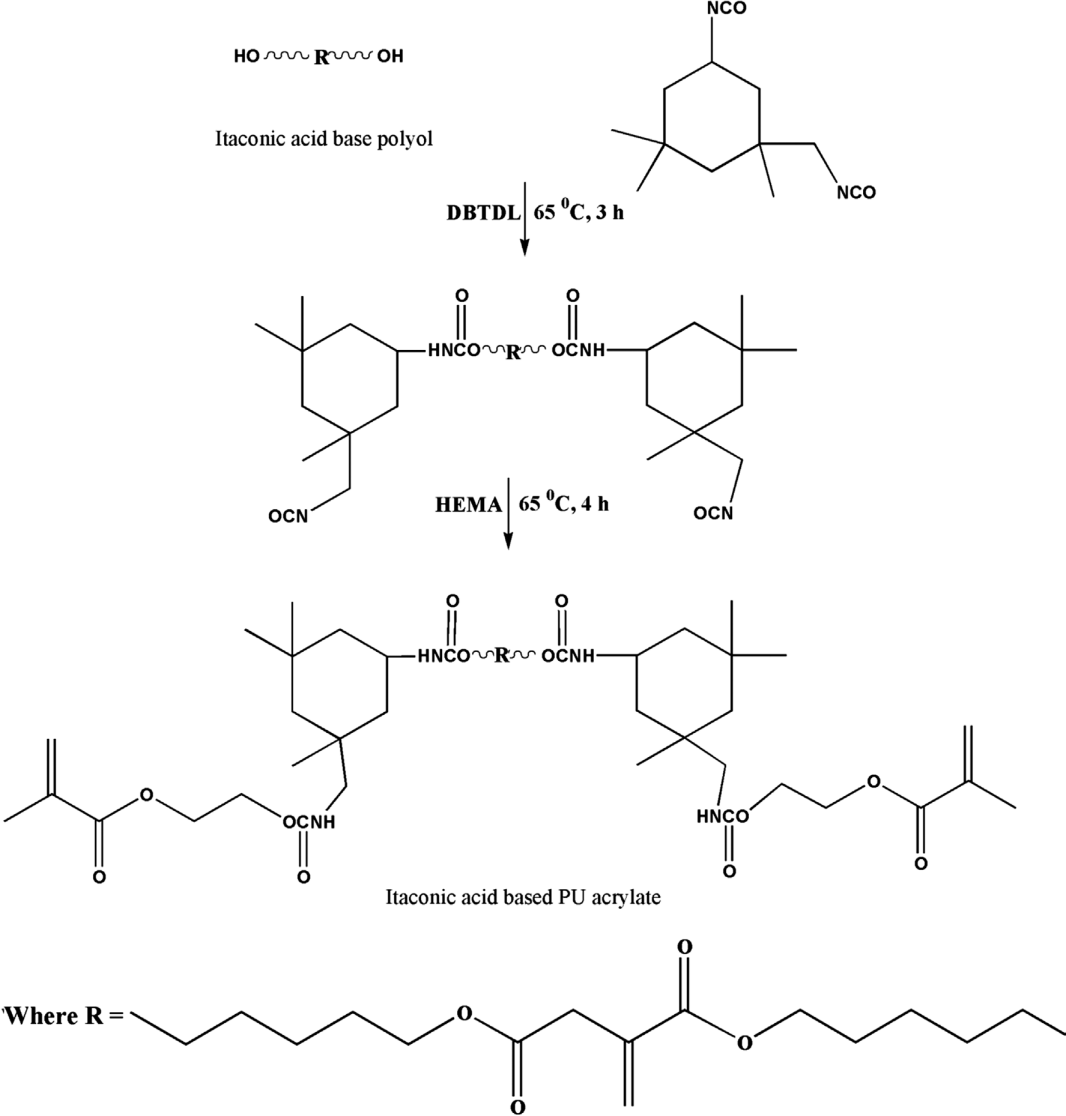
Synthesis route for UV curable PUA resin.

The behavior of coatings towards water is a most important property of coatings and which can determine their end application. The water absorption test was performed to evaluate the migration of water in cured coatings. The coatings were immersed in water for 24 h and the result is shown in Figure 11. The water absorption of UV curable PUA, PUA1, PUA2, PUA3 and PUA4 coatings is 0.95, 0.92, 0.87, 0.85 and 0.80 percents respectively. PUA4 coating show less water absorption than any other coating and it attributed to the highly cross-linked network. In addition, the water absorption of polymers is directly related to their free volume fraction and also to the presence of resin backbone. IA based PUA4 coating has aliphatic carbon chain backbone which not form any type of bond with water molecules and less free space for water absorption. Hence, IA based PUA4 coating has less affinity towards the water (Schemes 1 and 2).

### Chemical resistance

The cured coatings were immersed in 5% HCl and 5% NaOH solution for 24 h to evaluate the chemical resistance properties. It was observed for loss of gloss or adhesion after the completion of test. The acid and alkali test show no damages to the coating while both test show slight reduction in gloss. All UV cured PUA coatings are form highly crosslinked structure by reacting the unsaturation in UV irradiation and hence, coatings has resistance towards the acid and alkali. In addition, PUA coatings have polar N–H groups present in the backbone that creates hydrogen bonding in it or with other molecules and that can be considered effective physical barrier to acid and alkali solution.

### Solvent resistance

The solvent rub test conducted using methyl–ethyl ketone (MEK) and results revealed that the UV curable PUA coatings are sufficiently cross-linked. The high cross-linked networks have high resistance to the solvent rub and polar N–H groups of PUA coatings create a hydrogen bonding in it or with other molecules that enhance the solvent rub resistance properties.[42] From above study, it is concluded that all UV curable PUA coatings form sufficiently strong cross-linked network to resist the solvent.

### Mechanical properties

The UV curable PUA coatings are characterized for various mechanical properties and results are tabulated in Table 5. Scratch hardness tester checked the hardness of coatings and we observe excellent scratch hardness for UV curable conventional PUA and IA based PUA coatings. IA based PUA4 coating show better hardness properties as compared to the PUA, PUA1, PUA2 and PUA3 coatings. The observed results can be correlated to the cross-linked structure and strong crosslinking network which give good hardness properties.[41] IA based PUA resin have more reactive sites in the structure as compared to the conventional PUA resin and IA based PUA coatings form more crosslinked structure when undergoes UV curing. Hence, PUA4 coating showed high hardness value as compared to the PUA, PUA1, PUA2 and PUA3 coatings. All coatings show pencil hardness above 4H and this is due to the high crosslinking as discussed above. Coating adhesion with metal substrate was determined by crosscut test. The coatings show 100% adhesion to the metal substrates as measured by the cross-cut adhesion method. The UV curable PUA coatings have polar N–H groups present in the molecules that create hydrogen bond with metal substrate and improve the adhesion properties. Further, the flexibility of PUA, PUA1, PUA2, PUA3 and PUA4 coatings are observed to be excellent with no crack development during testing on conical mandrel apparatus. This can be attributed to the presence of long aliphatic chains and polar N–H groups along the chain length of PUA resin that creates hydrogen bonding in it or with other molecules.[42] In impact resistance test, all coatings have given good response to the high-speed load for intrusion impact test. This is due to the excellent balance maintained between the softer segments of long aliphatic chains of PUA and the hardness offered from high cross-linked structure of UV cured coatings. For extrusion impact test, all PUA coating show poor results as compared to the intrusion impact test. From the above study, UV curable IA based PUA coatings have shown better performance for all mechanical properties as compared to the conventional PUA coating.

**Table 5.** Mechanical properties of the UV-cured PUA coatings.

Properties		PUA	PUA1	PUA2	PUA3	PUA4
DFT (micron)		50–60	55–60	50–60	50–55	50–60
Gloss (60°)		112.2	104.9	101.2	102.3	101.4
Cross-cut adhesion		Pass	Pass	Pass	Pass	Pass
Pencil hardness		4H	4H	5H	6H	5H
Scratch hardness (g)		2100	2250	2200	2250	2350
Impact resistance	Intrusion	112	112	112	112	112
	Extrusion	60	64	68	74	72
Flexibility (mm)		0	0	0	0	0

## 6. Conclusion

The UV curable PUA resin is successfully synthesized from bio-based IA by simple condensation reaction in presence of pTSA catalyst. The IA based UV curable PUA resin synthesis is characterized by physicochemical analysis and further confirmed by FTIR, ^1^H and ^13^C-NMR spectroscopy. The synthesized IA based PUA resin incorporated in various concentrations into the conventional PUA and their effect on coating properties was studied. The DSC and TGA analysis show that thermal properties improve as concentration of IA based PUA resin increase in conventional PUA coatings. From XRD analysis, it is found that as concentration of IA based PUA in coatings increase; the coating becomes more rigid and crystalline. The UV curable IA based PUA coatings shows better mechanical, chemical resistance and solvent resistance properties as compared to the conventional PVA. From the above study, it is concluded that IA based UV curable PUA coating is good alternative to conventional PUA coating.

## Disclosure statement

No potential conflict of interest was reported by the authors.

## Funding

The work was supported by BRNS, DAE.
